# Assessment of Cerebral Hemodynamic Changes During Roll-Over Test in Healthy Pregnant Women and Those with Mild and Severe Preeclampsia

**DOI:** 10.3390/jcm14041182

**Published:** 2025-02-11

**Authors:** Dániel T. Nagy, Béla Fülesdi, Bence Kozma, Dénes Páll, Szilárd Szatmári, Petronella Hupuczi

**Affiliations:** 1 Department of Anesthesiology and Intensive Care, University of Debrecen, Nagyerdei krt. 98, H-4032 Debrecen, Hungary; nagy.daniel.tamas@gmail.com (D.T.N.); szatmari.szilard@gmail.com (S.S.); 2 Neuroscience Doctoral School, University of Debrecen, H-4032 Debrecen, Hungary; hupuczip@gmail.com; 3 Department of Obstetrics and Gynecology, Faculty of Medicine, University of Debrecen, Nagyerdei krt. 98, H-4032 Debrecen, Hungary; bence.kozma@med.unideb.hu; 4 Faculty of Medicine, Internal Medicine University of Debrecen, H-4032 Debrecen, Hungary; pall.denes@gmail.com; 5 Department of Medical Clinical Pharmacology, H-4032 Debrecen, Hungary; 6 Maternity Hospital Budapest, H-1126 Budapest, Hungary

**Keywords:** preeclampsia, roll over test, static cerebral autoregulation

## Abstract

**Background:** Preeclampsia (PE) and eclampsia are characterized by changes in cerebral hemodynamics, which may result in serious and even life-threatening neurological complications. The aim of the present work was to compare cerebral hemodynamic changes during the roll-over test in women with mild and severe PE. **Patients and methods:** Healthy pregnant and PE women in their third trimester were studied. Transcranial Doppler (TCD) measurements of the right middle cerebral artery (MCA) were performed in the left lateral position and 5 min after turning to the supine position (roll-over test = ROT). Besides cerebral blood flow measurements, the blood pressure was measured in the right arm using a standard mercury sphygmomanometer. An independent gynecologist categorized the preeclamptic patients into mild and severe groups based on the clinical and laboratory results. The TCD assessors were unaware of the patient grouping while performing the TCD and blood pressure measurements. **Results:** A total of 21 healthy pregnant females (mean age: 26.1 ± 5.1 yrs), 11 females with mild PE (28.2 ± 6.8 yrs), and 18 females with severe PE (29 ± 7.4 yrs) were studied. A significant increase in the mean arterial pressure was observed in all of the groups during the roll-over test: healthy pregnant patients: from 106.3 ± 16.3 to 113.8 ± 15.9 mmHg; patients with mild PE: from 100 ± 11.2 to 110 ± 8.7 mmHg; and patients with severe PE: from 106.3 ± 16.3 to 113.8 ± 15.8 mmHg. The MCA mean blood flow velocities in the left lateral position were significantly lower in the control patients than in those with PE: MCAV control: 71.2 ± 12.7 cm/s; mild PE: 78.2 ± 19.4 cm/s; and severe PE: 96 ± 15.6 cm/s, *p* < 0.001. Turning to the supine position resulted in a decrease in the MCAV in all of the groups, but the differences between the groups remained unchanged: controls: 69.5 ± 9.1 cm/s; mild PE: 75.7 ± 17.5 cm/s; and severe PE: 85.7 ± 18.4 cm/s, respectively, *p* = 0.014. A slight but statistically insignificant increase in the pulsatility index was observed in all of the groups. **Conclusions**: This is the first study comparing cerebral hemodynamic changes in healthy pregnant females and in those with mild and severe PE during a roll-over test. Changing the posture did not result in changes in the cerebral blood flow velocities in the healthy and preeclamptic pregnant patients. Our results indicate that static cerebral autoregulation is preserved both in the mild and severe preeclamptic patients.

## 1. Introduction

Preeclampsia occurs in 2–7% of healthy nulliparous women, but a higher incidence is reported in women with multifetal gestations and in those with a history of preeclampsia [1]. It is the most dangerous among the hypertensive disorders in pregnancy: based on epidemiologic data, preeclampsia is responsible for over 500,000 fetal and neonatal deaths and over 70,000 maternal deaths worldwide annually [2]. The importance of early recognition of the disease is proven by the fact that, in the case of preeclampsia being recognized in time and properly treated, the development of more serious forms can be prevented.

Preeclampsia is a multisystem disease that may result in various consequences, affecting the cardiovascular, cerebrovascular, and hemopoietic systems as well as hepatic and renal function. Recently, uteroplacental ischemia resulting in angiogenic imbalance has been implicated as the main pathophysiological background to explain these complications [3,4,5,6].

Cerebral autoregulation is the inherent ability of the brain circulation to keep cerebral blood flow constant during changes in the systemic blood pressure. It is believed that in preeclampsia, circulating factors that are released from the injured placenta may affect the endothelial function of the brain’s resistance arterioles and, thus, impair cerebral autoregulation [7]. Dynamic autoregulation describes the ability of the cerebral vasculature to react to sudden changes in the cerebral perfusion pressure, whereas static autoregulation reflects the autoregulatory ability in the resting state [8]. The impairment of dynamic cerebral autoregulation in preeclamptic pregnancies has been reported previously by several authors [9,10]. However, clinical data on alterations of static autoregulation in preeclamptic patients of different severities are scarce.

The aim of the present study was to assess whether static cerebral autoregulation is affected in mild and severe preeclamptic patients as compared to normal pregnant women. We hypothesized that preeclampsia may also result in impaired cerebral autoregulation, especially in its severe forms.

## 2. Methods

Healthy and preeclamptic patients of the Department of Obstetrics and Gynaecology, University of Debrecen, were included in this prospective, non-randomized cohort study. The study was approved by the Medical Ethics Committee of the University of Debrecen (code: 7056.RKEB, approval date: 27 June 2022) All patients gave written informed consent before participation. After explaining the investigation in detail, the subjects gave informed consent. Healthy pregnant women were outpatients with no previous or present signs of hypertension, diabetes mellitus, or significant renal disease in their history. The Department of Obstetrics and Gynecology, University of Debrecen, serves as a regional center for complicated pregnancies. Pregnant women with preeclampsia were selected by the obstetric outpatient department through regular checking of their blood pressure and proteinuria. Women who were considered high-risk were admitted to the Department of Obstetrics and Gynaecology, University of Debrecen, as inpatients. The following clinical data were recorded in all cases: age, parity, gestational age, previous history of preeclampsia or eclampsia, significant renal or cardiovascular disease, diabetes mellitus, or hypertension. Standard criteria were used for selecting the preeclamptic patients: at least 20 weeks’ gestational age, de novo hypertension, and blood pressure exceeding 140/90 mmHg on three different occasions, as well as proteinuria higher than 0.3 g/ day. Preeclamptic patients were grouped by an experienced obstetrician (BK) into mild and severe cases. Mild PE was defined as the presence of hypertension (blood pressure >140/90) on 2 occasions at least 6 h apart and mild proteinuria (>0.3 g/day), but without evidence of any organ damage in the patient. The definition of severe preeclampsia consisted of the following parameters: systolic blood pressure of 160 mm Hg or higher and/or diastolic blood pressure of 110 mm Hg or higher on 2 occasions at least 6 h apart and/or proteinuria of more than 5 g in a 24 h collection or more > 3 g on 2 random urine samples collected at least 4 h apart.

Roll-over test: Patients were asked to turn to their lateral decubitus position. After a 5 min of stabilization period, blood pressure was measured on the right arm using standard sphygmomanometer (OMRON BP5150 Bronze Upper Arm Blood Pressure Monitor, OMRON Ltd. Kyoto, Japan). Systolic, diastolic, and mean blood pressure values were registered. Thereafter, patients were asked to turn to supine position and after a 5 min stabilization period, blood pressure measurements were repeated. Because the mean determinant of the cerebral hemodynamic changes was the mean arterial pressure (MAP), only this value was taken into account in further analysis.

Transcranial Doppler measurements: The middle cerebral artery (MCA) was insonated using the 2 MHz sector probe of the Mindray M6 ultrasound device (Mindray, Shenzen, China) through the temporal bone. The middle cerebral artery was localized using the color mode of the device and blood flow velocities in the MCA were measured after angle correction. In the first stage of the roll-over test, the patients were asked to turn to the left lateral position. Consequently, proper placement of the transcranial color-coded duplex probe on the left side was not easy to perform and might have resulted in improper positioning. Because the right middle cerebral artery is accessible in both left lateral and supine positions, we decided to use the right side. Systolic, diastolic, and mean blood flow velocities and pulsatility indices were registered in the right middle cerebral artery both in the left lateral and in the supine position during the roll-over test by an experienced neurosonologist (BF). This person was not aware of the grouping status of the patients (healthy or preeclamptic).

Statistical analysis: In all datasets, normality tests were performed first. Normally distributed data are reported as means and standard deviations. Depending on the normality of the data, intragroup comparisons were either performed with paired t-tests (normally distributed values) or Mann–Whitney rank sum tests (values with non-normal distribution). Before–after blood pressure and blood flow velocity values that were measured during roll-over test in the different groups were compared either using the appropriate *t*-tests or using Kruskal–Wallis tests, depending on their distribution. The relationship between the percent change in mean arterial pressure during roll-over test and the contemporaneous percent change in the pulsatility index was assessed by Pearson’s test. A *p* < 0.05 value was accepted as a statistically significant difference.

## 3. Results

Twenty-one healthy pregnant and twenty-nine preeclamptic patients entered the study. The main clinical characteristics of the patients are summarized in Table 1. The selection of the preeclamptic patients based on the symptoms described in the methods section revealed 11 mild and 18 severe preeclamptic patients. There were no statistical differences among the three groups in terms of age, gestational age, and previous obstetrical history.

### 3.1. Mean Arterial Pressure Values During Roll-Over Test

**Figure 1:**
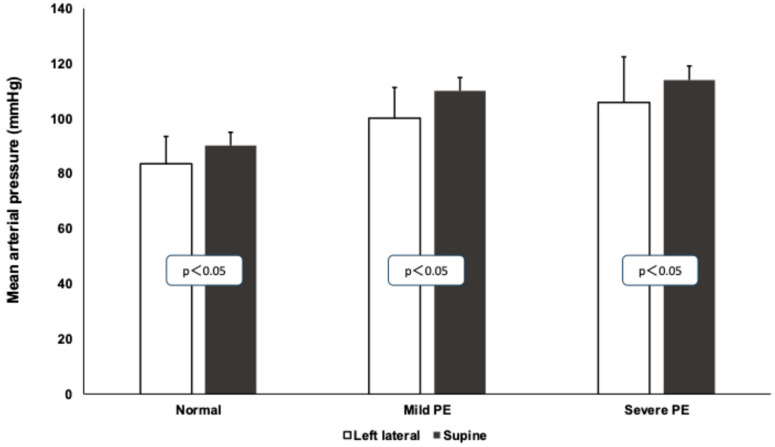
Mean arterial pressure values (in mmHg) in healthy pregnant and mild and severe preeclamptic patients during roll-over test. Means and standard deviations are presented. White columns represent left lateral, black columns represent the supine position.

A significant increase in the mean arterial pressure was observed in all of the groups during the roll-over test. Healthy pregnant patients: from 106.3 ± 16.3 to 113.8 ± 15.9 mmHg; patients with mild PE: from 100 ± 11.2 to 110 ± 8.7 mmHg; patients with severe PE: from 106.3 ± 16.3 to 113.8 ± 15.8 mmHg. The preeclamptic patients had higher blood pressures in both positions compared to the healthy patients (Figure 1).

### 3.2. Transcranial Doppler Measurements During Roll-Over Tests

**Figure 2:**
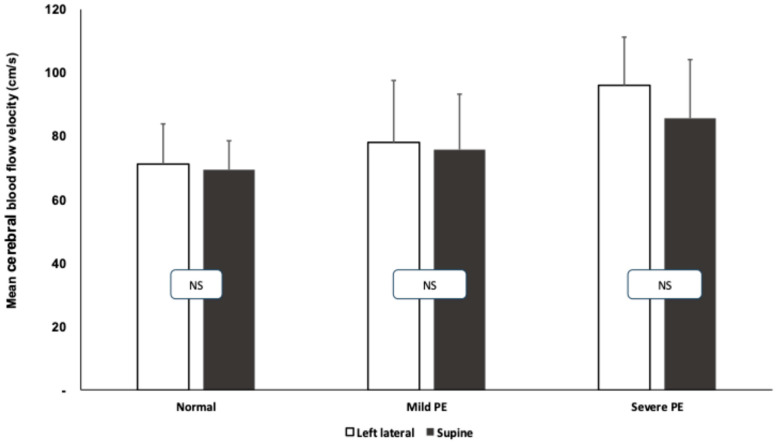
Mean blood flow velocity (cm/s) in the middle cerebral artery during roll-over test in healthy and preeclamptic patients. Means and standard deviations are presented. White columns represent left lateral, black columns represent the supine position.

The middle cerebral artery mean blood flow velocity was significantly higher in the resting state in the mild and severe preeclamptic patients compared to the healthy pregnant women (healthy: 67.4 ± 10.5 cm/s; mild PE: 75.3 ± 12.4; severe PE: 85.4 ± 13.1 cm/s). The results of the cerebral blood flow measurements during the roll-over tests are summarized in Figure 2. After turning from the left lateral to supine position, a slight but statistically insignificant change was observed in all of the groups, while the cerebral blood flow difference among the groups persisted (higher blood flow velocities in preeclamptic patients).

**Figure 3:**
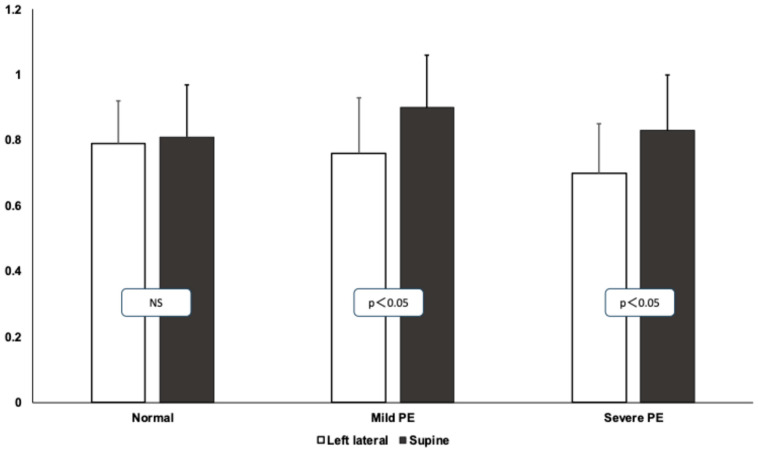
Changes in the pulsatility index during roll-over test in healthy and preeclamptic pregnant patients. Means and standard deviations are presented. White columns represent left lateral, black columns represent the supine position.

The pulsatility index values significantly increased in the preeclamptic patients while turning from the left lateral to supine position, indicating the vasoconstriction of the resistance arterioles of the brain circulation as a function of cerebral autoregulation. The results of the pulsatility indices are depicted in Figure 3.

The relationship between the percent change in the mean arterial pressure during the roll-over test and the contemporaneous percent change in the pulsatility index revealed a significant positive relationship between these two parameters in preeclamptic patients in general (Pearson’s correlation coefficient: 0.38; *p* = 0.012). When assessing the mild and severe cases separately, this relationship could also be demonstrated (Pearson’s correlation coefficients for mild PE: 0.34, *p* < 0.05; and for severe preeclampsia: 0.48, *p* < 0.01, respectively). This shows that the higher the increase in the mean arterial pressure during the roll-over test, the more the cerebral arterioles are constricted, indicating preserved cerebral autoregulation in the preeclamptic patients.

## 4. Discussion

In the present study, we used a physiological stimulus, the roll-over test [11], to assess cerebral blood flow alterations during systemic blood pressure changes. The test is based on the observation that turning from the left lateral to the supine position results in an increase in the blood pressure both in healthy pregnant women and those with preeclampsia, but the magnitude of blood pressure increase is higher in the preeclampsia group [12,13]. Although the discriminative power of the roll-over test in predicting preeclampsia is moderate [14], its non-invasive and physiological nature makes it an ideal stimulus for assessing cerebral autoregulation.

There are two basic concepts in the literature for explaining the pathophysiology of preeclampsia. According to the autoimmune theory, the initiating factor of the process is the materno-fetal (paternal) maladaptation of the immune system, resulting in an increased type 2 immune reaction towards antigens of paternal origin in the fetus [1]. It is conceived that this immune reaction results in the production of inflammatory mediators and they exert a generalized effect by activating the inflammatory system. According to the vascular theory, the first step in the development is an ischemic reperfusion injury of the placenta and this may lead to a generalized inflammatory reaction through the activation of the cytokine cascade. Although the two concepts represent two somewhat opposing schools of thought, the common feature in the pathophysiological concept is a generalized inflammatory reaction with endothelial activation and activation of the hemostatic and complement system [1,14,15]. Circulating inflammatory cytokines in preeclamptic patients are known to cause an imbalance between the endothelial factors that are responsible for vasodilation and vasoconstriction and are also capable of targeting the endothelial cells of the blood–brain barrier.

Physiologically, pregnancy causes a significant increase in the plasma volume and the cardiac output. The systemically increased blood flow is not equally distributed between the different organs. For instance, the uterine blood flow increases 10-fold by the end of the third trimester, while this increase in blood flow would be intolerable for the brain. Cerebral autoregulation is responsible for keeping the cerebral blood flow constant during systemic blood pressure changes. In normal pregnancy, a mild decrease or no change has been reported in the cerebral blood flow, despite the increased blood volume and cardiac output [16]. The main actors of cerebral autoregulation and vasoreactivity are cerebral arterioles of 200 µm in diameter. The reaction of these arterioles to metabolic (CO_2_ changes, O_2_ changes) or hemodynamic changes (cerebral autoregulatory reaction to changes in the cerebral perfusion pressure) occurs through endothelial NO/endothelin production, depending on the stimulus. The adaptation of the cerebral vascular bed to physiological changes during normal pregnancy also occurs through a balance between vasoconstrictor (i.e., endothelin) and vasodilatory (nitric oxide) endothelial factors, resulting in the physiological remodeling of the blood vessels [17]. As a consequence, in normal pregnancy, while the cerebral blood flow remains constant, the cerebral blood flow velocity decreases and cerebral vascular resistance increases along with advancing gestational age. In preeclampsia, however, the cerebral blood flow velocity in the cerebral arteries increases and the cerebral vascular resistance increases [8,18]. In preeclamptic patients, the altered function of the cerebral arterioles also causes the impaired vasoreactivity of the resistance arterioles during dynamic autoregulatory testing [19]. The importance of cerebral autoregulation testing is underlined by the fact that sudden increases in the systemic blood pressure in patients with impaired autoregulation may result in increased blood–brain barrier permeability, consequent microbleeds, and neuronal damage to the brain parenchyma. If the neuronal damage persists, after pathological pregnancies, the risk of Alzheimer’s disease and stroke is increased in later life or mild cognitive impairments may develop [8].

Cerebral autoregulation is usually tested by different methods that induce changes in the systemic blood pressure, such as the Valsalva maneuver, inducing lower body negative pressure, cuff inflation–deflation at lower extremities, head-up tilt table testing, and the administration of pharmacologic agents [8]. We decided to use the roll-over test because it is known that turning from the left lateral to supine position results in an increase in the mean arterial pressure in the third trimester. As compared to the other methods listed above, it was considered more of a physiological stimulus by us, because changing the body position may occur several times during the daily activity of the pregnant women. Furthermore, there were studies showing that a more than 20 mmHg elevation in the diastolic blood pressure may indicate preeclampsia [20]. Although later studies demonstrated a moderate sensitivity and positive predictive value of the roll-over test in preeclampsia [14,21], we used the ROT as a stimulus and not for prediction purposes.

The main difference between static and dynamic cerebral autoregulation is the dynamics of the response. As described above, the key actors of the autoregulatory reaction are the cerebral arterioles that are regulated by myogenic, neurogenic, and metabolic stimuli. It has been shown previously that the cerebral blood flow velocity decreases and the pulsatility index increases in the third trimester of normal pregnancy, indicating vasoconstriction of the cerebral arterioles [18,22,23,24]. Physiological hemodilution and hyperventilation may cause these changes in the third trimester. In contrast to this, in preeclamptic patients, the blood flow velocity is increased and the pulsatility index is decreased as a consequence of the decreased arteriolar tone due to the generalized endothelial dysfunction [18,22,23,24,25,26]. This alteration of the arteriolar tone does not affect the metabolic regulation of the cerebral arterioles, as was demonstrated by breath-holding and hyperventilation tests in preeclamptic pregnancies [27,28]. As dynamic autoregulation is impaired in preeclampsia, as has been shown by several authors [9,10,19,29], it is conceivable that during sudden alterations of the cerebral perfusion pressure, the cerebral arterioles are not capable of producing a timely autoregulatory response, but the slow vasodilatory and vasoconstrictor capacity of the cerebral arterioles are preserved.

The results of the present study indicate that static cerebral autoregulation is preserved in pregnant patients with mild and severe preeclampsia. We used 5 min resting periods for the measurement of the systemic blood pressure and cerebral blood flow velocities during the roll-over test; thus, our study can be considered as a static autoregulatory test. Turning from the left lateral to the supine position resulted in a significant increase in the systemic blood pressure in all of the groups and no significant changes could be observed in the mean blood flow velocities during the test in any of the groups, indicating preserved autoregulation. Meanwhile, the pulsatility indices increased in the mild and severe preeclamptic group, which refers to the vasoconstriction of the cerebral arterioles during turning from the left lateral to supine position.

Previous experimental data on static cerebral autoregulation in preeclamptic animals were inconclusive [7]. There were reports on impaired static autoregulation, but the majority of the studies described a shift of the autoregulatory curve toward higher blood pressure values [19]. Similarly to our results, Zatik and colleagues described an unaltered cerebral blood flow index in mild preeclamptic patients along with an increased resistance area product [22].

We have to mention the limitations of our study. This is a single-center, non-randomized case–control study and the number of included patients is limited. We may be criticized for the method for assessing cerebral blood flow. Blood flow velocities measured in the middle cerebral artery are not equal to but proportional to changes in the cerebral blood flow. Transcranial Doppler sonography has been widely used before to assess cerebral hemodynamics in pregnant patients. In the present study, we used the transcranial color-coded mode for vessel identification, which allows for angle correction and, thus, more precise blood flow velocity measurements. Due to the limited number of included patients, the results of the present study are mainly of pathophysiological value and do not allow one to draw clinical conclusions and the generalization of the findings is not possible.

In conclusion, our study indicates that static cerebral autoregulation is preserved in mild and severe preeclampsia. Further studies should delineate whether the combination of dynamic and static autoregulation tests is suitable for discriminating high-risk preeclamptic patients.

## Figures and Tables

**Table 1:** Clinical characteristics of the subjects. Means and standard deviations are presented. PE indicates preeclampsia; NA indicates non-applicable; H indicates hypertension; H + P indicates hypertension + proteinuria.

	Control (n = 21)	Mild PE (n = 11)	Severe PE (n = 18)
Age (years)	26.1 ± 5.6	28.2 ± 6.8	29.1 ± 7.4
Gestational age (weeks)	37.2 ± 3.1	32.7 ± 4.8	35.1 ± 3.4
Previous pregnancies (n)	1.5 (1–4)	1 (0–2)	1 (1–3)
Previous births (n)	1 (0–3)	0 (0–1)	1 (0–2)
Leading symptoms of PE	NA	H: 9 H + P: 2	H + P: 14H: 4

## Data Availability

Data are available from the corresponding author after reasonable request.
